# Dunglison’s Dictionary of Medical Science

**Published:** 1901-03

**Authors:** 


DUNGLISON'S DICTIONARY OF MEDICAL SCIENCE. Containing a full explanation of the various subjects and terms of Anatomy, Physiology, Medical Chemistry, Pharmacy, Pharmacology, Therapeutics, Medicine, Hygiene, Dietetics, Pathology, Surgery, Ophthalmology, Otology, Laryngology, Dermatology, Gynecology, Obstetrics, Pediatrics, Medical Jurisprudence, Dentistry, Veterinary Science, etc, etc. By ROBLEY DUNGLISON, M. D., LL. D., late Professor of the Institutes of Medicine in the Jefferson Medical College of Philadelphia. Edited by Richard J. Dunglison, A. M., M. D. New (22d) edition, thoroughly revised, greatly enlarged and improved, with the pronunciation, accentuation and derivation of the terms. In one imperial octavo volume of 1350 pages. Lea Brothers & Co., Philadelphia and New York. 1900. [Cloth, $7.00, net; full leather, $8.00 net.]

Dunglison's Medical Dictionary has again been revised and brought thoroughly to the latest date, with an appendix containing no less than 160 pages and 15,000 new words. The immense growth of medical terms and the necessity of an accurate knowledge of them is becoming increasingly important. For two generations Dunglison has been by common consent the standard authority. It has been used by tens of thousands of students in all English-speaking countries, and equally by practitioners. In the several revisions of the past decade about 65,000 new terms have been added, so that this standard authority may always be confidently consulted for the latest words.

All departments of medicine are represented, including veterinary and dental science, so that it is more complete and comprehensive than ever, and therefore liable to become more popular, if such a thing is possible. Many tables are incorporated in this edition, also much general information not usually found in medical dictionaries; for instance, the table of half-breeds, that on man, hair and so on, which are extremely interesting. The portrait of Robley Dunglison is a welcome addition, and the present author is to be thanked for giving the profession a chance to see the kindly face of the founder of this dictionary. In its new form it will creditably retain the position it has long held, as the authoritative lexicon of the medical sciences. It is heavily bound in leather, with a firm back and able to withstand good hard usage.—W. C. K.

